# Risk of Traumatic Injury in Patients With Early-Onset Parkinson's Disease: A Population-Based Matched Cohort Study

**DOI:** 10.1155/padi/6970763

**Published:** 2025-10-12

**Authors:** Takenori Akaike, Toshiki Fukasawa, Etsuro Nakanishi, Soichiro Masuda, Satomi Yoshida, Ryosuke Takahashi, Koji Kawakami

**Affiliations:** ^1^Department of Pharmacoepidemiology, Graduate School of Medicine and Public Health, Kyoto University, Kyoto, Japan; ^2^Japan Oncology Clinical Sciences Development, Oncology Therapeutic Area Unit, Takeda Pharmaceutical Company Limited, Osaka, Japan; ^3^Department of Neurology, Graduate School of Medicine, Kyoto University, Kyoto, Japan; ^4^Department of Clinical Medicine, Institute of Medicine, University of Tsukuba, Tsukuba, Japan; ^5^Kyoto University Office of Research Acceleration, Kyoto University, Kyoto, Japan

## Abstract

**Introduction:**

Early-onset Parkinson's disease (EOPD) shares similar clinical features to the late-onset form, but the risk of injury remains unclear. This study aimed to evaluate the risk of traumatic injury, including fracture, in patients with EOPD.

**Methods:**

This matched cohort study used a Japanese administrative claims database to compare the risk of traumatic injury and fracture between EOPD patients and the general population. EOPD was defined by diagnosis between ages 21 and 49 together with the initiation of anti-PD medication. Crude incidence rates and adjusted hazard ratios (aHRs) were estimated using Poisson and Cox regression models. Subgroup analyses were performed by age and sex.

**Results:**

In 368 EOPD patients and 1586 matched individuals from the general population, the traumatic injury rate was slightly higher in EOPD patients (9.5 vs. 7.9 events per 100 person-years), but the difference was not substantial (aHR, 1.2; 95% confidence interval [CI], 0.9–1.5). Fracture risk in the groups was similar, at 1.4 events per 100 person-years (aHR, 0.9; 95% CI, 0.5–1.6). Subgroup analyses showed an increased traumatic injury risk in EOPD patients aged 40–49 years (aHR, 1.4; 95% CI, 1.0–1.8) and in females (aHR, 1.3; 95% CI, 1.0–1.8). No clear differences were observed in other comparisons.

**Conclusion:**

No major difference in traumatic injury or fracture risk was found between EOPD patients and the general population. However, preventive interventions may be warranted for patients aged 40–49 years and for females due to their elevated injury risk.

## 1. Introduction

Early-onset Parkinson's disease (EOPD) has been defined as Parkinson's disease (PD) that begins between the ages of 21 and 40, and sometimes up to 50 years of age [1, 2]. The clinical presentation and pathogenesis of EOPD are generally similar to those of late-onset PD (LOPD) [1], except for a higher genetic predisposition, slower progression, and increased risk of levodopa-related complications [3]. The cardinal features of PD include tremor at rest, rigidity, bradykinesia, and postural instability [4]. As the disease progresses, it can lead to gait disturbances such as flexed posture and gait freezing, and thereby increase the risk of falls [4].

The incidence of falls in patients with PD is high. A systematic review reported that 35%–90% of patients experience at least one fall, and 18%–65% experience recurrent falls [5]. Such falls can result in injuries, fractures, and hospitalizations. Two population-based cohort studies showed an adjusted hazard ratio (aHR) of 2.25 for all types of fracture and 2.71 for hip fracture in patients with PD versus those without PD [6, 7]. However, to our knowledge, no studies have specifically investigated the risk of injury in EOPD patients. Here, we aimed to compare the incidence rates of injury in EOPD patients to those in the general population without EOPD.

## 2. Materials and Methods

### 2.1. Data Source

We used a Japanese administrative claims database provided by JMDC Inc. for the period from January 1, 2005, to October 31, 2023 [8–10]. As of March 2023, the database contains anonymized records from numerous corporate health insurance societies covering approximately 17 million employees and their dependents aged less than 75 years. It provides individual-level information on demographics, inpatient and outpatient diagnoses, and all reimbursed medical services, including procedures and pharmacy dispensing. Health checkup data are also available for a subset of individuals [11]. This study, along with the waiver of informed consent, was approved by the Ethics Committee of Kyoto University Graduate School and Faculty of Medicine (No. R4025), in accordance with Japanese ethical guidelines.

### 2.2. Study Design and Population

We conducted a matched cohort study which compared EOPD patients to reference individuals in the general population without EOPD. EOPD patients were defined as having an initial diagnosis of PD between the ages of 21 and 49 years and a first prescription for anti-PD medication within 60 days of the date of first diagnosis [12]. Cohort entry date was defined as the date of first diagnosis of PD and was limited to the period between April 1, 2012, and October 31, 2022 (1 year before October 31, 2023). The reason for limiting cohort entries to April 1, 2012, and later was that claims data from January 1, 2005, to March 31, 2012, documented the month only, and not the specific day. We included EOPD patients with at least 180 days of continuous enrollment in the database for the collection of baseline covariate data prior to cohort entry. We excluded patients with any of the following: history of traumatic injury, intentional self-harm, mental and behavioral disorders due to psychoactive substance use, or antipsychotic use during the 180 days before cohort entry; history of dementia, epilepsy, syncope, schizophrenia, mental retardation, attention-deficit/hyperactivity disorder, ataxia, hydrocephalus, ischemic and hemorrhagic stroke, Paget's disease of bone, rickets, osteomalacia, hyperparathyroidism, or secondary malignant neoplasm of bone and bone marrow before cohort entry [6, 9, 13]; or diagnosis of parkinsonism or drug-induced parkinsonism during the 5 years after cohort entry.

For each EOPD patient, up to five reference individuals were randomly selected from the database, matched on age, sex, period from database enrollment to cohort entry, and number of outpatient visits during the 180 days prior to the cohort entry date [14]. Reference individuals were allowed to be included multiple times across different matches [15]. To avoid selection bias due to differential loss to follow-up [16], each reference individual had to be continuously enrolled in the database until at least the date of first prescription of anti-PD medication in the corresponding EOPD patient [17]. Reference individuals were required to be free of EOPD at the cohort entry of their corresponding case but could be diagnosed with EOPD during follow-up (and would then stop contributing person-time to the reference group) [17]. The same exclusion criteria used for the EOPD group were applied to the reference group. Details of the inclusion and exclusion criteria can be found in Table S1 [18].

### 2.3. Study Outcomes and Follow-Up

The primary outcome was the incidence of traumatic injury including fractures. The secondary outcome was the incidence of fractures alone. Fractures were defined by a combination of International Classification of Diseases, Tenth Revision (ICD-10), codes and radiographic examination codes (Table S2). Injuries other than fractures were defined solely by ICD-10 codes [9].

The EOPD group and the reference group were followed for up to 5 years from cohort entry until the occurrence of a study outcome, death, disenrollment, or the end of the study period (October 31, 2023), whichever occurred first. If reference individuals developed PD, their follow-up was censored at the time of PD diagnosis.

### 2.4. Baseline Characteristics

The following baseline characteristics (Table S3) were assessed prior to cohort entry: (i) demographics (age and sex); (ii) cohort entry year; (iii) number of outpatient visits; (iv) body mass index (BMI); (v) exercise habits; (vi) comorbidities (diabetes and osteoporosis); and (vii) medications that could potentially impact fall-related injuries (antiarrhythmics, antidepressants, anxiolytics, corticosteroids, diuretics, hypnotics, and statins) [6, 7, 19].

### 2.5. Statistical Analysis

Imbalances in baseline characteristics between the comparison groups were assessed using standardized mean differences (SMDs). We estimated crude incidence rates, adjusted incidence rate differences (aIRDs), aHRs, and their corresponding 95% confidence intervals (CIs) for outcomes using Poisson and Cox regression models. Direct adjusted cumulative incidence curves were generated using Cox regression models [20]. All adjusted models accounted for matching factors, diabetes, and use of medications, including antidepressants, anxiolytics, hypnotics, and statins [15].

We also conducted subgroup analyses stratified by age (21–39 vs. 40–49 years) and sex to assess heterogeneity in the associations. Sensitivity analyses were performed in the overall population and the subgroups, using multiple imputations by chained equations to account for potential impact of missing data in BMI and exercise habits [21, 22]. On the assumption of missing at random, 50 iterated datasets were generated. In the analyses, BMI and exercise habits were added as covariates in the Poisson regression model for aIRD and the Cox regression model for aHR. The results from each imputed dataset were combined with Rubin's rules to obtain pooled estimates and their 95% CIs.

All analyses were conducted using SAS Version 9.4 (SAS Institute Inc.) and R Version 4.3.2 (R Foundation for Statistical Computing).

## 3. Results

### 3.1. Baseline Characteristics

**Figure 1:**
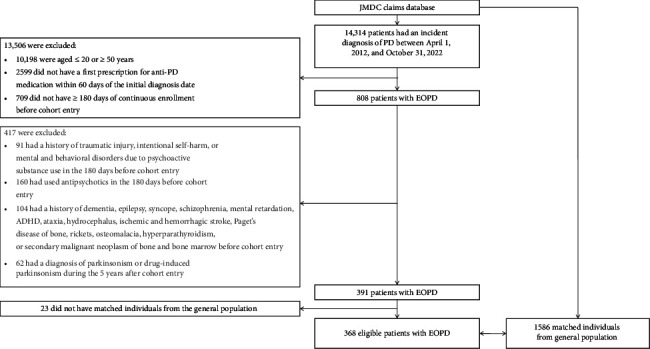
Flow diagram of the cohort study. Abbreviations: ADHD = attention-deficit/hyperactivity disorder, EOPD = early-onset Parkinson's disease, PD = Parkinson's disease.

The study cohort included 368 patients with EOPD (mean age, 39.7 years; 52.2% male) and 1586 reference individuals from the general population (mean age, 39.9 years; 52.6% male) (Figure 1 and Table 1). The majority of participants fell within the 40–49-year age group (61.1% of EOPD patients vs. 62.2% of reference individuals). Statin use was less common in the EOPD group (2.4% vs. 6.9%), whereas the use of antidepressants (11.1% vs. 4.4%), anxiolytics (17.4% vs. 6.5%), and hypnotics (13.0% vs. 6.7%) was more common. The median follow-up for traumatic injury was 2.6 years (interquartile range [IQR], 1.4–4.1) for EOPD patients and 2.8 years (IQR, 1.3–4.6) for reference individuals (Table S4).

### 3.2. Traumatic Injury and Fracture

During the five-year follow-up, 94 of the 368 patients with EOPD experienced traumatic injury (crude incidence rate, 9.5 events per 100 person-years; 95% CI, 7.6–11.4) compared with 358 of the 1586 reference individuals (crude incidence rate, 7.9 events per 100 person-years; 95% CI, 7.1–8.7). No clear difference was observed in traumatic injury risk (aIRD, 1.9 events per 100 person-years [95% CI, −0.9–4.8]; aHR, 1.2 [95% CI, 0.9–1.5]) (Table 2).

**Figure 2:**
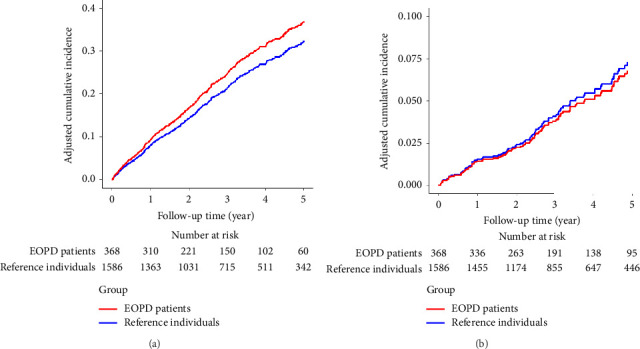
Adjusted cumulative incidence of (a) traumatic injury and (b) fracture in patients with early-onset Parkinson's disease and reference individuals. Abbreviation: EOPD = early-onset Parkinson's disease. Adjusted for matching factors (age, sex, period from database enrollment to cohort entry, and number of outpatient visits during the 180 days prior to the cohort entry date), diabetes, and use of antidepressants, anxiolytics, hypnotics, and statins.

Fracture risk was identical in both groups at 1.4 events per 100 person-years (95% CI, EOPD group: 0.7–2.1; reference group: 1.1–1.7), with an aIRD of −0.1 events per 100 person-years (95% CI, −0.9–0.7) and aHR of 0.9 (95% CI, 0.5–1.6). Adjusted cumulative incidence curves for traumatic injury and fracture are illustrated in Figure 2.

### 3.3. Subgroup Analysis

Subgroup analyses stratified by age and sex showed baseline characteristics similar to those of the overall population (Tables S5 and S6). Compared to the reference individuals, the aIRD and aHR for traumatic injuries among EOPD patients aged 21–39 years were −1.7 events per 100 person-years (95% CI, −5.6–2.0) and 0.8 (95% CI, 0.5–1.3), respectively, and 0.0 events per 100 person-years (95% CI, −1.0–1.0) and 1.0 (95% CI, 0.4–2.5) for fractures (Table 2). In contrast, EOPD patients aged 40–49 years had an increased rate of traumatic injuries (aIRD, 4.0 events per 100 person-years [95% CI, 0.0–8.0]; aHR, 1.4 [95% CI, 1.0–1.8]) but showed no substantial difference in fracture rate (aIRD, −0.2 events per 100 person-years [95% CI, −1.1–0.8]; aHR, 0.9 [95% CI, 0.4–1.8]).

When stratified by sex, female patients had a slightly higher traumatic injury rate than males, although their 95% CIs overlapped. In females, the aIRD for traumatic injury was 3.5 events per 100 person-years (95% CI, −1.4–8.3), with an aHR of 1.3 (95% CI, 1.0–1.8), compared to an aIRD of 0.7 events per 100 person-years (95% CI, −2.9–4.4) and aHR of 1.1 (95% CI, 0.8–1.5) for males. The aIRD for fracture in females was 0.7 events per 100 person-years (95% CI, −1.7–3.2) versus −0.6 events per 100 person-years (95% CI, −1.7–0.4) in males, with corresponding aHRs of 1.3 (95% CI, 0.6–2.6) and 0.6 (95% CI, 0.3–1.4), respectively. Adjusted cumulative incidence curves for each subgroup are shown in Figures S1 and S2.

### 3.4. Sensitivity Analysis

Sensitivity analyses involving imputed values for BMI and exercise habits as covariates yielded results consistent with the primary and secondary outcomes. In the overall population, the aIRD and aHR for traumatic injury were 2.4 (95% CI, −1.0–5.9) and 1.2 (95% CI, 0.9–1.5); the aIRD and aHR for fracture were −0.1 (95% CI, −1.0–0.7) and 0.9 (95% CI, 0.5–1.6), respectively (Table 3). Subgroup analyses stratified by age and sex also showed consistent results in the sensitivity analyses.

## 4. Discussion

Our matched cohort study found that traumatic injury and fracture rates in patients with EOPD were not substantially different from those in the general population. However, subgroup analyses revealed a higher traumatic injury rate among patients with EOPD aged 40–49 years and among female patients, whereas fracture rates remained comparable across all subgroups. These findings remained consistent and robust across sensitivity analyses using multiple imputations by chained equations to account for the potential impact of missing data in BMI and exercise habits.

Two previous meta-analyses reported elevated fracture risks at multiple bone sites among patients with LOPD. Specifically, one meta-analysis estimated a pooled hazard ratio of 3.13 (95% CI, 2.53–3.87) for hip fractures [23], while the other found a relative risk of 2.40 (95% CI, 2.04–2.82) for hip fractures and 1.80 (95% CI, 1.60–2.01) for nonvertebral fractures [24]. In contrast, our study did not show an increase in traumatic injury or fracture rates among EOPD patients overall, with an aHR of 1.2 (95% CI, 0.9–1.5) for traumatic injury and 0.9 (95% CI, 0.5–1.6) for fracture. These findings highlight the differential risk profiles between EOPD and LOPD populations. Unlike LOPD, EOPD patients often have a slower progression of motor symptoms (e.g., tremor, muscle rigidity, and postural instability), which may help preserve motor function, leading to fewer falls and lower risk of injuries [3, 25]. Furthermore, osteoporosis is frequently observed in LOPD patients and is associated with reduced mobility and lower BMI [26]. However, the EOPD group did not show a higher prevalence of osteoporosis, decreased BMI, or reduced exercise habits compared with reference individuals (Table 1). These factors may explain why no clear differences in traumatic injury or fracture rates were observed in EOPD patients. In the subgroups with higher injury rates (i.e., patients aged 40–49, female patients), the fracture risks did not differ. Bone mineral density (BMD)—a widely used measure of bone health—peaks around the age of 20 years and then gradually declines with age [27]. Given that EOPD population is relatively young (21–49 years), their BMDs remain near peak levels, which likely prevents fractures even if falls occur. Thus, although those subgroups experienced more injuries, their bones were not more fragile than those of the general population, and their fracture risks did not increase. Moreover, EOPD generally maintains better motor function and balance, which may result in less severe falls that cause minor injuries rather than fractures. This aligns with the observed association between injury and fracture risks within the subgroups.

In subgroup analyses, EOPD patients aged 40–49 years showed an increased rate of traumatic injury, whereas no differences were found among those aged 21–39 years, indicating the importance of age in injury risk for PD patients [28]. The elevated injury risk observed in the 40–49 age group may be explained by two main considerations. One is the potential influence of PD medication. For example, dopamine agonists may be associated with an increased risk of falls, possibly due to side effects such as sudden uncontrollable somnolence and increased freezing frequency [29]. Treatment strategies often differ by patient age [30, 31], which could be one of the factors contributing to the elevated injury risk in EOPD patients aged 40–49 years. Another is an age-related PD factor. Older onset PD is associated with earlier emergence of balance problems and a higher probability of fall, and different ages at onset vary in motor phenotype such as tremor dominant versus postural instability and gait difficulty dominant types [30, 32–34]. Age at onset may contribute to the injury risk in this subgroup. Even minor injuries, primarily due to falls, often provoke a fear of falling and subsequent reduction in physical activity, which in turn initiates a negative cascade of decreased mobility, further loss of muscle strength, impaired balance, and associated diseases such as constipation, cardiovascular problems, and osteoporosis. These consequences are likely to jeopardize quality of life and independence and ultimately increase healthcare costs [35, 36]. Hence, our findings suggest that early preventive interventions could benefit this age group. Such strategies may include muscle strengthening, balance and gait training through physical therapy, environment hazard assessment at home or in the community, screening for risk factors such as history of prior fracture and BMD, and careful review of PD therapy as well as medications with sedative or hypotensive effects. For fall management in LOPD, an expert panel recommends first identifying the specific type of fall and then tailoring screening and treatment accordingly [36]. This approach may also apply to EOPD, given that both forms of PD share most clinical features.

Subgroup analysis by sex indicated a slightly higher rate of traumatic injury and fracture in female EOPD patients than males, although the 95% CIs overlapped. Females are more prone to falls and experience more rapid age-related bone loss, leading to higher osteoporosis prevalence [36, 37]. Hormonal influences, including estrogen decline during menopause, are known to increase the risk of osteoporosis and fractures, particularly in females with PD. Although most female patients in our cohort were likely premenopausal, a subset may have experienced menopause during follow-up, potentially contributing to the risk elevation [23]. While poorer adherence to a certain PD medication in females may be indicated [38], in general, sex differences in overall treatment adherence have not been fully elucidated, and the potential impact of such differences on the injury and fracture risk remains unknown [39, 40]. Other sex-specific factors may involve motor symptoms such as greater postural instability and more levodopa-induced dyskinesia as well as disparities such as a longer time from onset of symptoms to diagnosis, and access to specialist care or multiprofessional treatment services in females, compared to males [41, 42]. These factors may contribute to these elevated injury and fracture risks, consistent with existing studies on fracture risk in PD populations [6, 23].

This study should be interpreted in light of several limitations. First, some misclassification of PD diagnosis may remain, although we strengthened diagnostic specificity by combining ICD-10 codes with anti-PD medications and excluding potential drug-induced parkinsonism. Second, our outcome assessment relied solely on ICD-10 codes for traumatic injuries, whereas fracture identification required both ICD-10 codes and radiographic examination codes, potentially affecting accuracy. Third, the administrative claims database we used lacked certain clinical information, such as PD-related manifestations and BMD. Although we performed comprehensive adjustment for covariates, the influence of these unmeasured factors as confounders cannot be ruled out. Fourth, despite our use of a large database, the study may have been underpowered to detect differences in traumatic injury risk because of the small sample size and the rarity of outcome events. Finally, as our study focused on a Japanese population, the transportability of our findings to other populations may be limited.

## 5. Conclusion

In conclusion, this population-based matched cohort study found no major difference in traumatic injury or fracture rates between EOPD patients and the general population. However, the higher traumatic injury rate observed among EOPD patients aged 40–49 years and among females indicates a need for targeted preventive strategies in these subgroups.

## Figures and Tables

**Table 1:** Baseline characteristics of patients with early-onset Parkinson's disease and reference individuals.

	EOPD patients (*n* = 368)	Reference individuals (*n* = 1586)	SMD
Age (years), mean (SD)	39.7 (7.8)	39.9 (7.7)	0.026
Age group			0.021
21–29 years	54 (14.7)	226 (14.2)	
30–39 years	89 (24.2)	374 (23.6)	
40–49 years	225 (61.1)	986 (62.2)	
Male	192 (52.2)	834 (52.6)	0.008
Cohort entry year			0.067
2012	4 (1.1)	17 (1.1)	
2013	7 (1.9)	32 (2.0)	
2014	15 (4.1)	55 (3.5)	
2015	12 (3.3)	58 (3.7)	
2016	27 (7.3)	117 (7.4)	
2017	39 (10.6)	152 (9.6)	
2018	42 (11.4)	200 (12.6)	
2019	58 (15.8)	258 (16.3)	
2020	53 (14.4)	236 (14.9)	
2021	64 (17.4)	265 (16.7)	
2022	47 (12.8)	196 (12.4)	
No. of outpatient visits, mean (SD)	6.3 (6.1)	5.6 (5.2)	0.121
BMI (kg/m^2^), mean (SD)	22.8 (3.9)	23.7 (4.4)	0.198
Missing	147 (39.9)	559 (35.2)	
Exercise habits			0.131
Yes	26 (7.1)	167 (10.5)	
No	169 (45.9)	736 (46.4)	
Missing	173 (47.0)	683 (43.1)	
Comorbidities			
Diabetes	14 (3.8)	130 (8.2)	0.186
Osteoporosis	7 (1.9)	15 (0.9)	0.081
Medication use			
Antiarrhythmics	37 (10.1)	183 (11.5)	0.048
Antidepressants	41 (11.1)	70 (4.4)	0.253
Anxiolytics	64 (17.4)	103 (6.5)	0.341
Corticosteroids	27 (7.3)	120 (7.6)	0.009
Diuretics	12 (3.3)	70 (4.4)	0.060
Hypnotics	48 (13.0)	106 (6.7)	0.215
Statins	9 (2.4)	109 (6.9)	0.211

*Note:* Data are presented as number (percentage) of individuals unless otherwise indicated.

Abbreviations: BMI = body mass index, EOPD = early-onset Parkinson's disease, SD = standard deviation, SMD = standardized mean difference.

**Table 2:** Crude incidence rates, adjusted incidence rate differences, and adjusted hazard ratios for study outcomes.

	Patients, n	Person-years, n	Events, n	Crude incidence rate per 100 person-years (95% CI)	aIRD per 100 person-years (95% CI)^a^	aHR (95% CI)^a^
Traumatic injury						
EOPD patients	368	989	94	9.5 (7.6–11.4)	1.9 (−0.9–4.8)	1.2 (0.9–1.5)
Reference individuals	1586	4540	358	7.9 (7.1–8.7)	Reference	Reference
Fracture						
EOPD patients	368	1158	16	1.4 (0.7–2.1)	−0.1 (−0.9–0.7)	0.9 (0.5–1.6)
Reference individuals	1586	5115	72	1.4 (1.1–1.7)	Reference	Reference
Subgroup analysis						
21–39 years						
Traumatic injury						
EOPD patients	143	361	26	7.2 (4.4–10.0)	−1.7 (−5.6–2.0)	0.8 (0.5–1.3)
Reference individuals	600	1621	131	8.1 (6.7–9.5)	Reference	Reference
Fracture						
EOPD patients	143	398	6	1.5 (0.3–2.7)	0.0 (−1.0–1.0)	1.0 (0.4–2.5)
Reference individuals	600	1827	27	1.5 (0.9–2.0)	Reference	Reference
40–49 years						
Traumatic injury						
EOPD patients	225	628	68	10.8 (8.2–13.4)	4.0 (0.0–8.0)	1.4 (1.0–1.8)
Reference individuals	986	2919	277	7.8 (6.8–8.8)	Reference	Reference
Fracture						
EOPD patients	225	760	10	1.3 (0.5–2.1)	−0.2 (−1.1–0.8)	0.9 (0.4–1.8)
Reference individuals	986	3288	45	1.4 (1.0–1.8)	Reference	Reference
Male						
Traumatic injury						
EOPD patients	192	537	44	8.2 (5.8–10.6)	0.7 (−2.9–4.4)	1.1 (0.8–1.5)
Reference individuals	834	2404	178	7.4 (6.3–8.5)	Reference	Reference
Fracture						
EOPD patients	192	615	6	1.0 (0.2–1.8)	−0.6 (−1.7–0.4)	0.6 (0.3–1.4)
Reference individuals	834	2697	39	1.4 (1.0–1.9)	Reference	Reference
Female						
Traumatic injury						
EOPD patients	176	452	50	11.1 (8.0–14.1)	3.5 (−1.4–8.3)	1.3 (1.0–1.8)
Reference individuals	752	2136	180	8.4 (7.2–9.7)	Reference	Reference
Fracture						
EOPD patients	176	544	10	1.8 (0.7–3.0)	0.7 (−1.7–3.2)	1.3 (0.6–2.6)
Reference individuals	752	2418	33	1.4 (0.9–1.8)	Reference	Reference

Abbreviations: aHR = adjusted hazard ratio, aIRD = adjusted incidence rate difference, CI = confidence interval, EOPD = early-onset Parkinson's disease.

^a^Adjusted for matching factors, diabetes, and use of antidepressants, anxiolytics, hypnotics, and statins. In the subgroup analysis of individuals aged 21–39 years, statins were excluded from the covariates, and in the analysis of fractures in the female subgroup, antidepressants were excluded.

**Table 3:** Adjusted incidence rate differences, and adjusted hazard ratios for sensitivity analyses.

EOPD patients vs. reference individuals	aIRD per 100 person-years (95% CI)^a^	aHR (95% CI)^a^
Traumatic injury	2.4 (−1.0–5.9)	1.2 (0.9–1.5)
Fracture	−0.1 (−1.0–0.7)	0.9 (0.5–1.6)
Subgroup analysis		
21–39 years		
Traumatic injury	−2.1 (−6.5–2.2)	0.8 (0.5–1.3)
Fracture	0.0 (−1.0–0.9)	1.0 (0.4–2.4)
40–49 years		
Traumatic injury	5.0 (0.6–9.9)	1.4 (1.0–1.8)
Fracture	−0.2 (−1.3–1.0)	0.9 (0.4–1.8)
Male		
Traumatic injury	1.3 (−3.1–5.7)	1.2 (0.8–1.6)
Fracture	−0.7 (−1.9–0.5)	0.6 (0.3–1.5)
Female		
Traumatic injury	4.0 (−1.8–9.7)	1.3 (0.9–1.8)
Fracture	0.6 (−1.7–3.0)	1.3 (0.6–2.6)

Abbreviations: aHR = adjusted hazard ratio, aIRD = adjusted incidence rate difference, CI = confidence interval.

^a^Adjusted for matching factors, BMI, exercise habits, diabetes, and use of antidepressants, anxiolytics, hypnotics, and statins. In the subgroup analysis of individuals aged 21–39 years, statins were excluded from the covariates, and in the analysis of fractures in the female subgroup, antidepressants were excluded.

## Data Availability

The data cannot be shared publicly due to the privacy policy of JMDC Inc.

## Supporting Information

Table S1: Definitions of inclusion and exclusion criteria. Table S2: Definitions of outcomes. Table S3: Definitions of baseline characteristics. Table S4: Follow-up and reasons for censor. Table S5: Baseline characteristics of patients with early-onset Parkinson's disease and reference individuals, stratified by age. Table S6: Baseline characteristics of patients with early-onset Parkinson's disease and reference individuals, stratified by sex. Figure S1: Adjusted cumulative incidence of traumatic injury by age and sex. Figure S2: Adjusted cumulative incidence of fracture by age and sex.
