# Supplementary material for: Risk of Traumatic Injury in Patients With Early-Onset Parkinson's Disease: A Population-Based Matched Cohort Study
Source: Parkinsons Dis. 2025 Oct 12;2025:6970763. doi: 10.1155/padi/6970763 (PMC12535810; doi:10.1155/padi/6970763)
Supplement: Supporting Information — Additional supporting information can be found online in the Supporting Information section. [file 6970763.f1.docx]

**Supplementary Material**

**Risk of traumatic injury in patients with early-onset Parkinson’s disease: A population-based matched cohort study**

Takenori Akaike, Toshiki Fukasawa, Etsuro Nakanishi, Soichiro Masuda, Satomi Yoshida, Ryosuke Takahashi, Koji Kawakami

**Table of Contents**

[Table S1. Definitions of inclusion and exclusion criteria 2](#_Toc188707754)

[Table S2. Definitions of outcomes 4](#_Toc188707755)

[Table S3. Definitions of baseline characteristics 5](#_Toc188707756)

[Table S4. Follow-up and reasons for censor 6](#_Toc188707757)

[Table S5. Baseline characteristics of patients with early-onset Parkinson’s disease and reference individuals, stratified by age 7](#_Toc188707758)

[Table S6. Baseline characteristics of patients with early-onset Parkinson’s disease and reference individuals, stratified by sex 9](#_Toc188707759)

[Fig. S1. Adjusted cumulative incidence of traumatic injury by age and sex. 11](#_Toc188707760)

[Fig. S2. Adjusted cumulative incidence of fracture by age and sex. 12](#_Toc188707761)

# Table S1. Definitions of inclusion and exclusion criteria

| **Inclusion criteria** | **Definitions** | **Assessment period (day)** ^a^ |
| --- | --- | --- |
| PD | ICD-10 code (standard disease code): G20 (3320002, 8842319, 8843950, 8843951, 8843952, 8843953, 8843954, 8846156, 8843850, 8843851, 8843852, 8843853, 8843854, 8846157, 8846158, 8846159) | [−∞, 0] |
| Anti-PD medication | ATC code: N04BA, N04BC, N04BD | [0, 60] |
| Cohort entry date | Date of first diagnosis of PD is defined as the cohort entry date. At least 180 days of continuous enrollment in the database before cohort entry is required. | [April 1, 2012, October 31, 2022] |
| **Exclusion criteria** | **Definitions** | **Assessment period (day)** ^a^ |
| Traumatic injury | ICD-10 code: S00–T14 | [−180, 0] |
| Intentional self-harm | ICD-10 code: X60–X84, T14.9 | [−180, 0] |
| Mental and behavioral disorders due to psychoactive substance use | ICD-10 code: F10–F19 | [−180, 0] |
| Antipsychotics | ATC code: N05A | [−180, 0] |
| Dementia ^b^ | ICD-10 code (standard disease code): F00–F03, F06.7, F07.2 (8831776, 8843158, 8843267, 8843268, 8843535), G20 (8850077), G30, G31.0–G31.8, G31.9 (3319002, 8849351) | [−∞, 0] |
| Epilepsy | ICD-10 code: G40–G41  -and-  ATC code: N03A, N05BA | [−∞, 0] |
| Syncope | ICD-10 code (standard disease code): R55, R56.8 (7803001, 7803024, 7803012, 7817002, 3459009, 3451014, 7803027, 7803002, 3451009, 7803021, 7803009) | [−∞, 0] |
| Schizophrenia ^b^ | ICD-10 code: F20 | [−∞, 0] |
| Mental retardation ^b^ | ICD-10 code: F70–F79 | [−∞, 0] |
| ADHD ^b^ | ICD-10 code: F90, F98.8 | [−∞, 0] |
| Ataxia ^b^ | ICD-10 code: G10, G11, G13, R27, G31.2, G31.9, A81 | [−∞, 0] |
| Hydrocephalus ^b^ | ICD-10 code: G91, Q03, Q05.4 | [−∞, 0] |
| Ischemic and hemorrhagic stroke ^c^ | ICD-10 code: I60–I64, I69 | [−∞, 0] |
| Paget’s disease of bone ^b^ | ICD-10 code: M88 | [−∞, 0] |
| Rickets ^b^ | ICD-10 code (standard disease code): E55.0, E83.3 (8839503, 8833309, 8850116, 8850120), N25.0 (8835613) | [−∞, 0] |
| Osteomalacia ^b^ | ICD-10 code: M83 | [−∞, 0] |
| Hyperparathyroidism ^b^ | ICD-10 code (standard disease code): E21 (8844594, 2520003, 2520001, 2520010), N25.8 (8847502, 8844495) | [−∞, 0] |
| Secondary malignant neoplasm of bone and bone marrow ^b^ | ICD-10 code: C79.5 | [−∞, 0] |
| Parkinsonism or drug-induced parkinsonism | ICD-10 code (standard disease code): G20 (3320001, 8830558, 8841415), G21 | [0, end of follow-up] |

Abbreviations: ADHD, attention-deficit/hyperactivity disorder; ATC, Anatomical Therapeutic Chemical; ICD-10, International Classification of Diseases, Tenth Revision; PD, Parkinson’s disease.

^a^ The cohort entry date was set as day 0. Inclusion and exclusion criteria were evaluated during the assessment period.

^b^ At least two claims recorded more than 30 days apart.

^c^ At least one inpatient claim or a minimum of two outpatient claims recorded more than 30 days apart.

# Table S2. Definitions of outcomes

| **Outcomes** | **Definitions** |
| --- | --- |
| Traumatic injury | ICD-10 code: S00–T14 |
| Fracture | Fracture events were defined by claims using a combination of ICD-10 codes and radiographic examination codes within seven days after the diagnosis date.  ICD-10 code: S02, S12, S22, S32, S42, S52, S62, S72, S82, S92, T02  -and-  Radiographic examination code: 170000210, 170000310, 170016010, 170016910, 170017010, 170020270, 170024470, 170024910, 170025010, 170025210, 170025310, 170025510, 170025710, 170025810, 170025910, 170026110, 170026310, 170028810, 170035810, 170036010, 170000410, 170000510, 170000610, 170000730, 170000810, 170022730, 170022830, 170022930, 170001910, 170002010, 170002110, 170005530, 170005630, 170006930, 170007030, 170027910, 170028010, 170028110, 170029330, 170029430, 170029530, 170029630, 170031350, 170032050, 170032150, 170032250, 170032650, 170007130, 170007230, 170023950, 170032350, 170021550, 170021750, 170023750, 170024250, 170031550 |

Abbreviation: ICD-10, International Classification of Diseases, Tenth Revision.

# Table S3. Definitions of baseline characteristics

| **Baseline characteristics** | **Definitions** | **Assessment period (day)** ^a^ |
| --- | --- | --- |
| Age (years) | — | [0, 0] |
| Sex | Male, female | [0, 0] |
| Cohort entry year | 2012–2022 | [0, 0] |
| No. of outpatient visits | — | [−180, 0] |
| BMI (kg/m^2^) | — | [−365, 0] |
| Exercise habits ^b^ | Yes, no, missing | [−365, 0] |
| Comorbidities |  |  |
| Diabetes ^c^ | ICD-10 code: E10–E14 | [−180, 0] |
| Osteoporosis | ICD-10 code: M80, M81 | [−180, 0] |
| Medication use |  |  |
| Antiarrhythmics | ATC code: C01B, C07AA07, C07AB09, C07AB14, C08DB01, C08DA01 | [−180, 0] |
| Antidepressants | ATC code: N06A | [−180, 0] |
| Anxiolytics | ATC code: N05B | [−180, 0] |
| Corticosteroids | Defined as a prednisolone-equivalent dose of at least 5 mg per day.  ATC code: H02AB | [−180, 0] |
| Diuretics | ATC code: C03CA01, C03CA04 | [−180, 0] |
| Hypnotics | ATC code: N05C | [−180, 0] |
| Statins | ATC code: C10AA | [−180, 0] |

Abbreviations: ATC, Anatomical Therapeutic Chemical; BMI, body mass index; ICD-10, International Classification of Diseases, Tenth Revision.

^a^ The cohort entry date was set as day 0. Baseline characteristics were evaluated during the assessment period.

^b^ Engages in exercise for thirty minutes or more, at least two days a week, for over a year.

^c^ At least one inpatient claim or a minimum of two outpatient claims recorded more than 30 days apart.

# Table S4. Follow-up and reasons for censor

|  | **EOPD patients**  **(n = 368)** | **Reference individuals**  **(n = 1,586)** |
| --- | --- | --- |
| **Traumatic injury** |  |  |
| Follow-up (years), median (IQR) | 2.6 (1.4, 4.1) | 2.8 (1.3, 4.6) |
| Reason for censor |  |  |
| Traumatic injury | 94 (25.5) | 358 (22.6) |
| Death | 0 | 3 (0.2) |
| Parkinson’s disease | NA | 2 (0.1) |
| Disenrollment | 76 (20.7) | 266 (16.8) |
| End of the study period (October 31, 2023) | 138 (37.5) | 619 (39.0) |
| End of the five-year follow-up | 60 (16.3) | 338 (21.3) |
| **Fracture** |  |  |
| Follow-up (years), median (IQR) | 3.1 (1.8, 5.0) | 3.3 (2.0, 5.0) |
| Reason for censor, n (%) |  |  |
| Fracture | 16 (4.4) | 72 (4.5) |
| Death | 0 | 3 (0.2) |
| Parkinson’s disease | NA | 2 (0.1) |
| Disenrollment | 88 (23.9) | 304 (19.2) |
| End of the study period (October 31, 2023) | 169 (45.9) | 764 (48.2) |
| End of the five-year follow-up | 95 (25.8) | 441 (27.8) |

Abbreviations: EOPD, early-onset Parkinson’s disease; IQR, interquartile range.

*Note:* Data are presented as number (percentage) of individuals unless otherwise indicated.

# Table S5. Baseline characteristics of patients with early-onset Parkinson’s disease and reference individuals, stratified by age

|  | 21–39 years | | | 40–49 years | | |
| --- | --- | --- | --- | --- | --- | --- |
|  | EOPD patients  (n = 143) | Reference individuals  (n = 600) | SMD | EOPD patients  (n = 225) | Reference individuals  (n = 986) | SMD |
| Age (years), mean (SD) | 31.5 (5.5) | 31.5 (5.3) | 0.009 | 45.0 (3.0) | 45.1 (3.0) | 0.025 |
| Male | 66 (46.2) | 284 (47.3) | 0.024 | 126 (56.0) | 550 (55.8) | 0.004 |
| Cohort entry year |  |  | 0.088 |  |  | 0.080 |
| 2012 | 3 (2.1) | 15 (2.5) |  | 1 (0.4) | 2 (0.2) |  |
| 2013 | 6 (4.2) | 28 (4.7) |  | 1 (0.4) | 4 (0.4) |  |
| 2014 | 6 (4.2) | 20 (3.3) |  | 9 (4.0) | 35 (3.5) |  |
| 2015 | 5 (3.5) | 24 (4.0) |  | 7 (3.1) | 34 (3.4) |  |
| 2016 | 9 (6.3) | 33 (5.5) |  | 18 (8.0) | 84 (8.5) |  |
| 2017 | 17 (11.9) | 67 (11.2) |  | 22 (9.8) | 85 (8.6) |  |
| 2018 | 7 (4.9) | 34 (5.7) |  | 35 (15.6) | 166 (16.8) |  |
| 2019 | 22 (15.4) | 94 (15.7) |  | 36 (16.0) | 164 (16.6) |  |
| 2020 | 23 (16.1) | 102 (17.0) |  | 30 (13.3) | 134 (13.6) |  |
| 2021 | 30 (21.0) | 119 (19.8) |  | 34 (15.1) | 146 (14.8) |  |
| 2022 | 15 (10.5) | 64 (10.7) |  | 32 (14.2) | 132 (13.4) |  |
| No. of outpatient visits, mean (SD) | 5.4 (5.1) | 4.8 (4.4) | 0.134 | 6.9 (6.6) | 6.2 (5.5) | 0.121 |
| BMI (kg/m^2^), mean (SD) | 21.0 (3.0) | 22.5 (4.2) | 0.418 | 23.4 (4.0) | 24.1 (4.5) | 0.149 |
| Missing | 88 (61.5) | 330 (55.0) |  | 59 (26.2) | 229 (23.2) |  |
| Exercise habits |  |  | 0.149 |  |  | 0.165 |
| Yes | 7 (4.9) | 33 (5.5) |  | 19 (8.4) | 134 (13.6) |  |
| No | 36 (25.2) | 189 (31.5) |  | 133 (59.1) | 547 (55.5) |  |
| Missing | 100 (69.9) | 378 (63.0) |  | 73 (32.4) | 305 (30.9) |  |
| Comorbidities |  |  |  |  |  |  |
| Diabetes | 3 (2.1) | 29 (4.8) | 0.150 | 11 (4.9) | 101 (10.2) | 0.204 |
| Osteoporosis | 3 (2.1) | 4 (0.7) | 0.123 | 4 (1.8) | 11 (1.1) | 0.055 |
| Medication use |  |  |  |  |  |  |
| Antiarrhythmics | 12 (8.4) | 53 (8.8) | 0.016 | 25 (11.1) | 130 (13.2) | 0.064 |
| Antidepressants | 19 (13.3) | 23 (3.8) | 0.343 | 22 (9.8) | 47 (4.8) | 0.194 |
| Anxiolytics | 24 (16.8) | 32 (5.3) | 0.371 | 40 (17.8) | 71 (7.2) | 0.324 |
| Corticosteroids | 7 (4.9) | 48 (8.0) | 0.127 | 20 (8.9) | 72 (7.3) | 0.058 |
| Diuretics | 4 (2.8) | 10 (1.7) | 0.077 | 8 (3.6) | 60 (6.1) | 0.118 |
| Hypnotics | 23 (16.1) | 25 (4.2) | 0.403 | 25 (11.1) | 81 (8.2) | 0.098 |
| Statins | 0 | 16 (2.7) | 0.234 | 9 (4.0) | 93 (9.4) | 0.218 |

Abbreviations: BMI, body mass index; EOPD, early-onset Parkinson’s disease; SD, standard deviation; SMD, standardized mean difference.

*Note:* Data are presented as number (percentage) of individuals unless otherwise indicated.

# Table S6. Baseline characteristics of patients with early-onset Parkinson’s disease and reference individuals, stratified by sex

|  | Male | | | Female | | |
| --- | --- | --- | --- | --- | --- | --- |
|  | EOPD patients  (n = 192) | Reference individuals  (n = 834) | SMD | EOPD patients  (n = 176) | Reference individuals  (n = 752) | SMD |
| Age (years), mean (SD) | 40.5 (7.1) | 40.7 (7.0) | 0.017 | 38.8 (8.4) | 39.1 (8.3) | 0.034 |
| Age group |  |  | 0.007 |  |  | 0.035 |
| 21–29 years | 23 (12.0) | 99 (11.9) |  | 31 (17.6) | 127 (16.9) |  |
| 30–39 years | 43 (22.4) | 185 (22.2) |  | 46 (26.1) | 189 (25.1) |  |
| 40–49 years | 126 (65.6) | 550 (65.9) |  | 99 (56.2) | 436 (58.0) |  |
| Cohort entry year |  |  | 0.067 |  |  | 0.084 |
| 2012 | 4 (2.1) | 17 (2.0) |  | 0 | 0 |  |
| 2013 | 3 (1.6) | 13 (1.6) |  | 4 (2.3) | 19 (2.5) |  |
| 2014 | 4 (2.1) | 16 (1.9) |  | 11 (6.2) | 39 (5.2) |  |
| 2015 | 8 (4.2) | 38 (4.6) |  | 4 (2.3) | 20 (2.7) |  |
| 2016 | 14 (7.3) | 67 (8.0) |  | 13 (7.4) | 50 (6.6) |  |
| 2017 | 17 (8.9) | 67 (8.0) |  | 22 (12.5) | 85 (11.3) |  |
| 2018 | 21 (10.9) | 100 (12.0) |  | 21 (11.9) | 100 (13.3) |  |
| 2019 | 26 (13.5) | 116 (13.9) |  | 32 (18.2) | 142 (18.9) |  |
| 2020 | 32 (16.7) | 142 (17.0) |  | 21 (11.9) | 94 (12.5) |  |
| 2021 | 37 (19.3) | 148 (17.7) |  | 27 (15.3) | 117 (15.6) |  |
| 2022 | 26 (13.5) | 110 (13.2) |  | 21 (11.9) | 86 (11.4) |  |
| No. of outpatient visits, mean (SD) | 6.2 (6.6) | 5.5 (5.3) | 0.123 | 6.4 (5.5) | 5.8 (5.0) | 0.119 |
| BMI (kg/m^2^), mean (SD) | 23.5 (3.5) | 24.8 (4.4) | 0.338 | 21.7 (4.4) | 21.8 (3.8) | 0.018 |
| Missing | 53 (27.6) | 203 (24.3) |  | 94 (53.4) | 356 (47.3) |  |
| Exercise habits |  |  | 0.201 |  |  | 0.124 |
| Yes | 17 (8.9) | 128 (15.3) |  | 9 (5.1) | 39 (5.2) |  |
| No | 107 (55.7) | 427 (51.2) |  | 62 (35.2) | 309 (41.1) |  |
| Missing | 68 (35.4) | 279 (33.5) |  | 105 (59.7) | 404 (53.7) |  |
| Comorbidities |  |  |  |  |  |  |
| Diabetes | 7 (3.6) | 100 (12.0) | 0.315 | 7 (4.0) | 30 (4.0) | 0.001 |
| Osteoporosis | 3 (1.6) | 4 (0.5) | 0.108 | 4 (2.3) | 11 (1.5) | 0.060 |
| Medication use |  |  |  |  |  |  |
| Antiarrhythmics | 20 (10.4) | 103 (12.4) | 0.061 | 17 (9.7) | 80 (10.6) | 0.032 |
| Antidepressants | 22 (11.5) | 45 (5.4) | 0.220 | 19 (10.8) | 25 (3.3) | 0.295 |
| Anxiolytics | 31 (16.1) | 57 (6.8) | 0.295 | 33 (18.8) | 46 (6.1) | 0.390 |
| Corticosteroids | 14 (7.3) | 56 (6.7) | 0.023 | 13 (7.4) | 64 (8.5) | 0.042 |
| Diuretics | 8 (4.2) | 45 (5.4) | 0.058 | 4 (2.3) | 25 (3.3) | 0.064 |
| Hypnotics | 27 (14.1) | 63 (7.6) | 0.211 | 21 (11.9) | 43 (5.7) | 0.220 |
| Statins | 7 (3.6) | 88 (10.6) | 0.271 | 2 (1.1) | 21 (2.8) | 0.120 |

Abbreviations: BMI, body mass index; EOPD, early-onset Parkinson’s disease; SD, standard deviation; SMD, standardized mean difference.

*Note:* Data are presented as number (percentage) of individuals unless otherwise indicated.


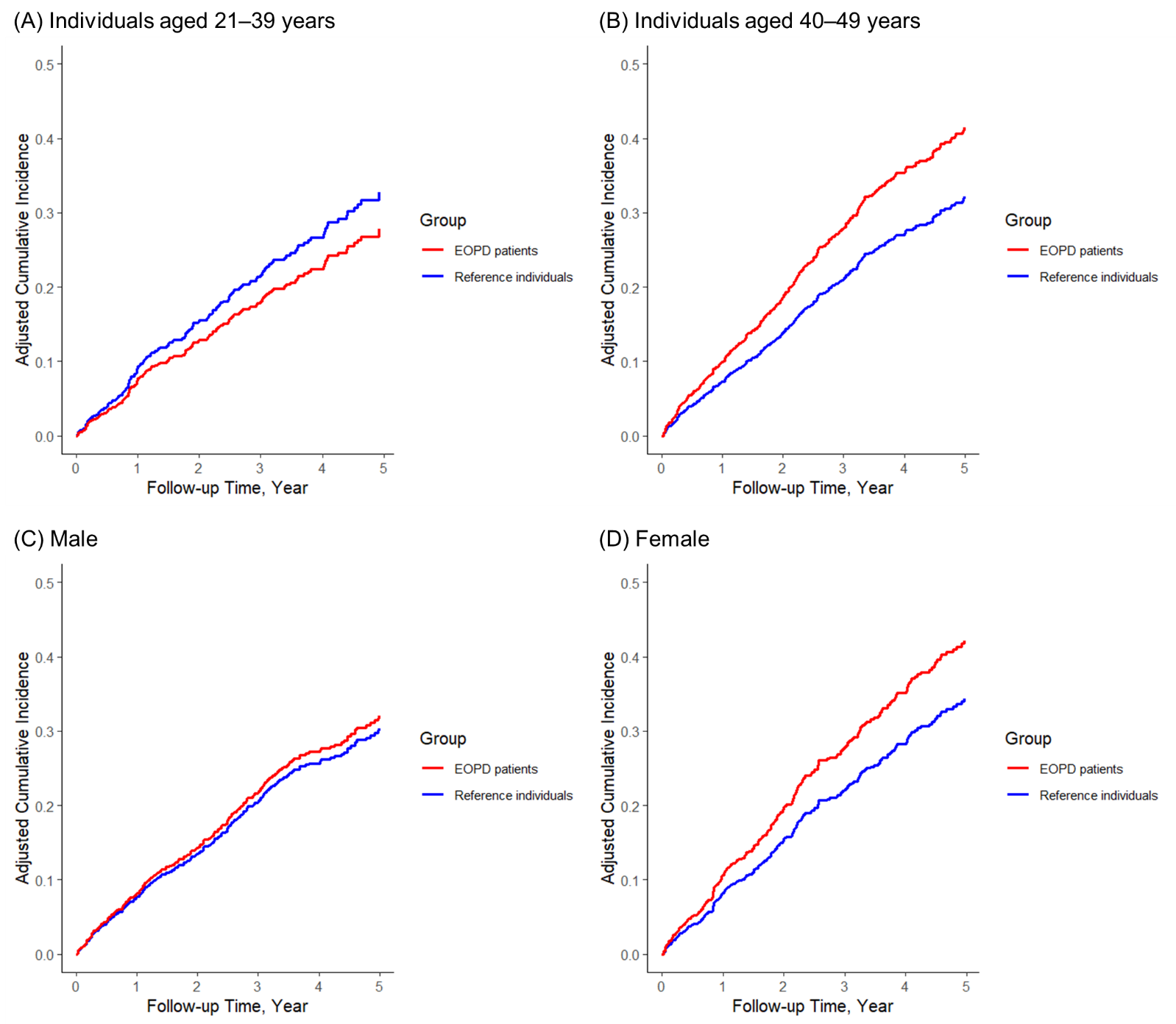


# Fig. S1. Adjusted cumulative incidence of traumatic injury by age and sex.

(A) Individuals aged 21–39 years; (B) individuals aged 40–49 years; (C) males; and (D) females.

Abbreviation: EOPD, early‑onset Parkinson’s disease.


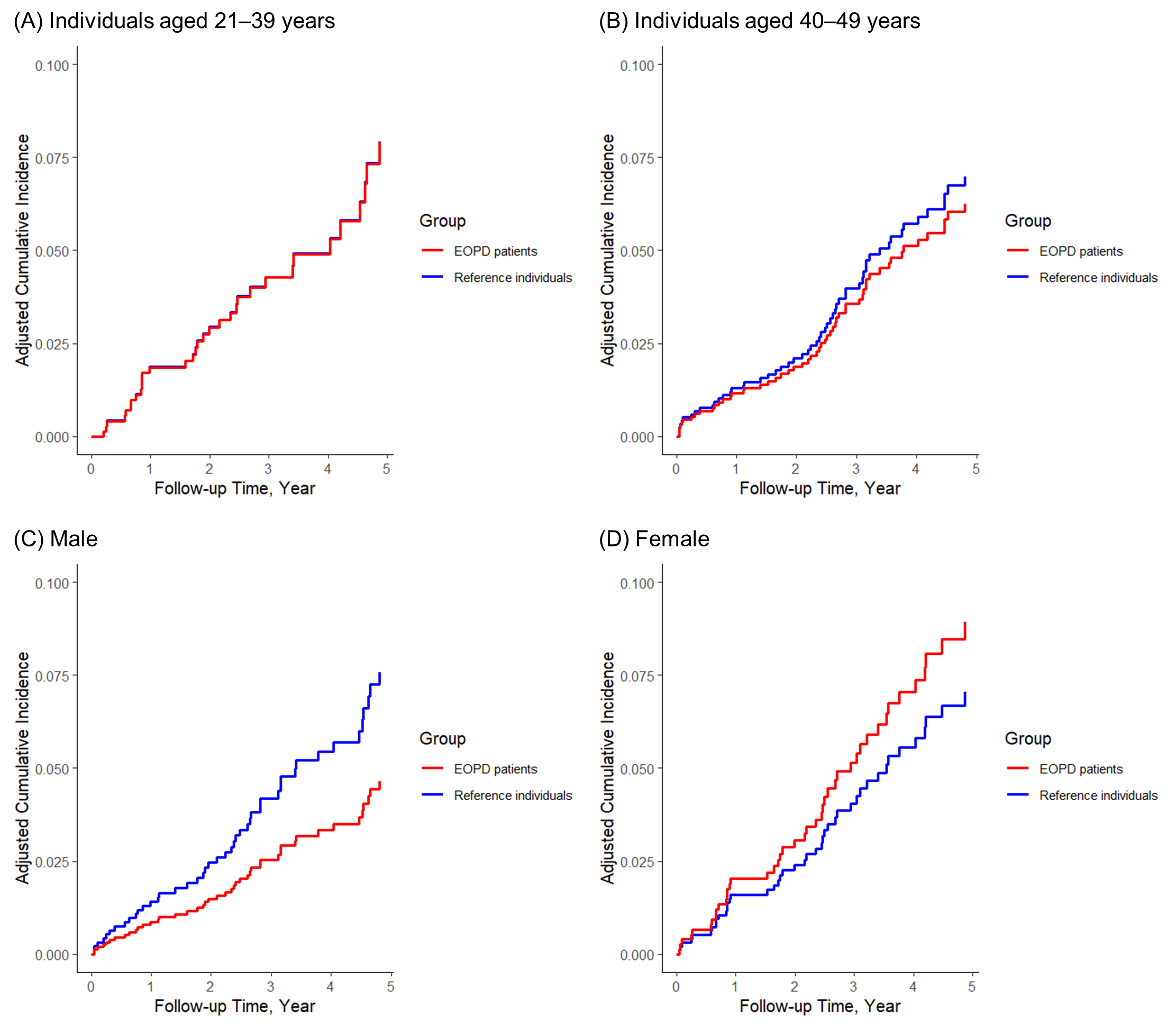


# Fig. S2. Adjusted cumulative incidence of fracture by age and sex.

(A) Individuals aged 21–39 years; (B) individuals aged 40–49 years; (C) males; and (D) females.

Abbreviation: EOPD, early‑onset Parkinson’s disease.
